# Regulation of the HIV-1 promoter by HIF-1α and Vpr proteins

**DOI:** 10.1186/1743-422X-8-477

**Published:** 2011-10-24

**Authors:** Satish L Deshmane, Shohreh Amini, Satarupa Sen, Kamel Khalili, Bassel E Sawaya

**Affiliations:** 1 Center for Neurovirology, Department of Neuroscience, Temple University School of Medicine, 3500 N. Broad Street, Philadelphia, PA 19140, USA; 2 Molecular Studies of Neurodegenerative Diseases Laboratory - Department of Neurology, Temple University School of Medicine, 3420 N. Broad Street, Philadelphia, PA 19140, USA; 3 Department of Biology, College of Science and Technology, Temple University, 1900 N. 12th Street, Philadelphia, PA 19122, USA

## Abstract

We previously demonstrated the ability of HIV-1 Vpr protein to activate the oxidative stress pathway, thus leading to the induction of the hypoxia inducible factor 1 alpha (HIF-1α). Therefore, we sought to examine the interplay between the two proteins and the impact of HIF-1α activation on HIV-1 transcription. Using transient transfection assays, we identified the optimal concentration of HIF-1α necessary for the activation of the HIV-1 promoter as well as the domain within HIF-1α responsible for this activation. Our findings indicated that activation of the HIV-1 LTR by Vpr is HIF-1α dependent. Furthermore, we showed that both Vpr and HIF-1α activate the HIV-1 promoter through the GC-rich binding domain within the LTR. Taken together, these data shed more light on the mechanisms used by Vpr to activate the HIV-1 promoter and placed HIF-1α as a major participant in this activation.

## Introduction

The HIV-1 accessory protein viral protein R (Vpr) is synthesized late in the HIV-1 life cycle, packaged into the virion, and is essential for HIV-1 replication in macrophages [1]. The infected macrophages have been shown to release Vpr, which affects neuronal growth and plasticity [2]. Studies from several groups have demonstrated that Vpr mediates multiple functions, including nuclear import of the HIV-1 pre-integration complex [3], G_2 _cell cycle arrest, transactivation of both viral replication and host genes and induction of apoptosis [4]. Most of these Vpr functions have been confirmed in an *in vitro *system. However, the physiological effect of Vpr *in vivo*, especially in neurons as well as its concentration [5], remains to be identified. Vpr was also detected in a soluble form in Cerebrospinal fluid (CSF) and sera of HIV-1-infected patients displaying neurological disorders [2]. Vpr was shown to activate the production and accumulation of the reactive oxygen species (ROS), a phenomenon that led to the activation of the hypoxia inducible factor 1 alpha (HIF-1α) [6].

Reactive oxygen species (ROS), especially superoxide anions (O_2_^-^) and hydrogen peroxide (H_2_O_2_), have been shown to promote HIF-1α protein accumulation *in vitro *and *in vivo *[7]. ROS also play an important role in the pathophysiology of many diseases such as atherosclerosis, tumor progression, Alzheimer's disease, Parkinson's disease, and amyotrophic lateral sclerosis [8-10]. Hypoxia on the other hand, has been shown to be a key component of the tumor microenvironment, causing genetic instability by affecting the DNA repair capacity of tumor cells [11]. Hypoxia is also known to increase angiogenesis, to up regulate glycolytic enzymes and to provide selective pressure for cancer cell-survival [12].

HIF-1 is a heterodimeric-transcription factor consisting of two distinct components, the oxygen-sensitive α subunit (HIF-1α) and constitutively expressed β subunit [HIF-1β, also known as the acryl hydrocarbon receptor nuclear translocator (ARNT)], both of which are members of the PAS (Per/ARNT/Sim) protein family [13,14]. HIF-1α binds to HIF-1β and induces hypoxic gene expression [15]. Under normoxic conditions, HIF-1α protein is barely detectable because it is rapidly decayed. The HIF-1α structural features and their known biological functions are as follows: The N-terminal domain of HIF-1α contains a basic helix-loop-helix domain and a PAS domain that are essential for dimerization with HIF-1β and binding to the conserved HIF-response element (HRE) DNA sequence (5'-RCGTG-3') [16]. The C-terminal domain of HIF-1α contains several regulatory domains responsible for oxygen-dependent gene expression. These include two separate transactivation domains, N-TAD (N-terminal transactivation domain; residues 531-575) and C-TAD (C-terminal transactivation domain; residues 786-826), which bind general transcriptional co activators such as CBP/p300, SRC-1, and TIF-2 [17]. Two transactivation domains flank an inhibitory domain (residues 576- 785), and a nuclear localization signal, located at both N-and C-terminal domains (residues 17-74 and residues 718-721, respectively). The oxygen dependent destruction domain (ODD, residues 401-603) is a critical component of HIF-1α and is involved in HIF-1α protein stability. The ODD contains two PEST-like motifs at residues 499-518 and 581-600, which may also regulate HIF-1α protein degradation.

In this manuscript, we demonstrated the presence of a functional interplay between Vpr and HIF-1α leading to the activation of the LTR. Further, our findings indicate that Vpr's ability to activate the LTR is HIF-1α dependent.

## Results and discussion

**Figure 1:**
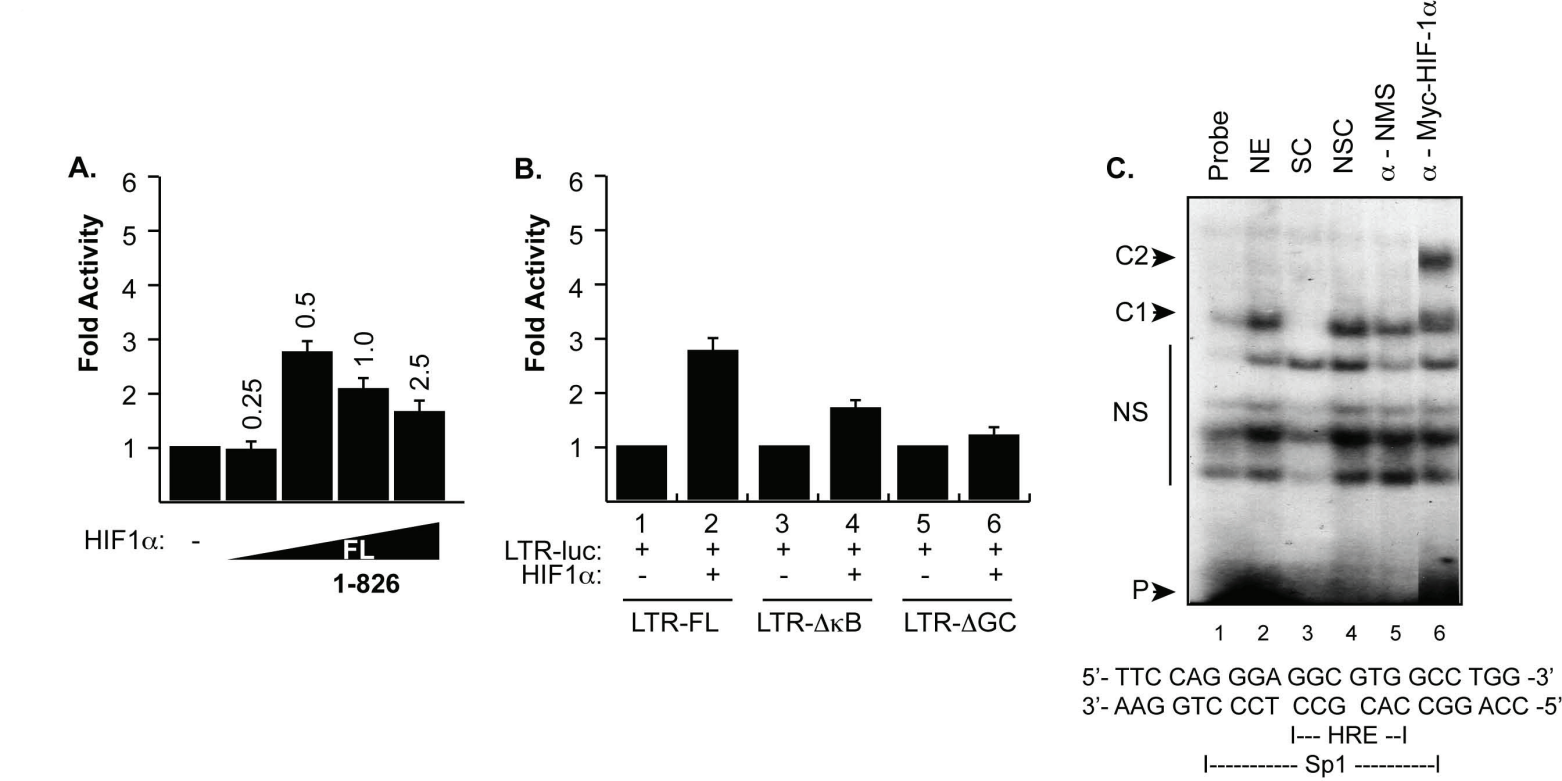
**Effect of HIF-1α on HIV-1 LTR**. ***A ***and ***B***. 786-O cells were transfected with 0.1 μg of the LTR-Luc full length or deletion mutants along with an increasing concentration of HIF-1α (A) or 0.5 μg of HIF-1α (B). The amount of DNA in each transfection mixture was normalized with pcDNA_6_HisA. Luciferase activity was determined 48 hours after transfection. Results are displayed as histograms. ***C***. Approximately 100, 000 cpm of synthetic [γ^32^P]-labeled double-stranded DNA oligonucleotide probe corresponding to the HIV-1 LTR GC-rich site was incubated with 10 μg of nuclear extracts prepared from microglial cells transfected with c-myc-HIF-1α. Labeled probe was also incubated with nuclear extracts prepared from pcDNA-transfected microglial cells in the presence of a specific DNA competitor (cold probe, lane 3), non-specific competitor (an unrelated dsDNA) (lane 4), anti-c-myc antibody (lane 6) and normal mouse serum (NMS) (lane 5).

First, we sought to identify the optimal concentration of HIF-1α needed for the activation of the HIV-1 promoter. Human kidney cells, 786-O, which do not express endogenous HIF-1α [18] (1 × 10^5^) were transfected with a reporter plasmid containing the LTR upstream regulatory sequence fused to luciferase reporter gene (0.1 μg) together with an increasing concentration of HIF-1α. Twenty-four hours post transfection, the cells were washed and processed for luciferase assay. As shown in Figure 1A, 0.5 μg of HIF-1α was the optimal concentration to activate the LTR.

Activation of the LTR by HIF-1α provided us with the rationale to identify the *cis*-element within the LTR responsible for this activation. 786-O cells were transfected with 0.5 μg of HIF-1α expression plasmid together with reporter plasmids containing the LTR full length or its deletion mutants where the κB or the GC-rich motifs were removed, respectively. These motifs were shown to be the binding sites for NF-κB and Sp1 transcription factors. As shown in Figure 1B, HIF-1α activates the LTR and its mutant derivative (LTR-ΔκB), however the activation was altered when the GC-rich motif was removed. The HIV-1 LTR has been shown to contain three GC-rich motifs localized between positions -78 and -42 [19]. Our findings led us to conclude that HIF-1α activates the LTR through the GC-rich motif. Interestingly, several factors have been also shown to activate the HIV-1 LTR through their interplay with Sp1 including the HIV-1 Vpr [20].

Since HIF-1α activates the HIV-1 LTR, we next investigated that potential mechanism involved in such activation. Therefore, we examined if HIF-1α can activate the LTR directly since the LTR contains a predicted HRE located between -71 and -67 [*RCGTG*, where R could be an A or G]. We performed gel shift assays using nuclear extracts derived from microglia, which we transfected with 5 μg c-*myc*-HIF-1α expression plasmids. Ten micrograms of nuclear extract prepared from microglial cells were incubated with labeled GC-rich dsDNA primer spanning nucleotides -82 to -61. As shown in Figure 1C, the intensity of the band corresponding to the DNA-HIF-1α complex was enhanced in cells transfected with a plasmid expressing HIF-1α (complex C1 in lane 2). To examine whether HIF-1α protein formed complex C1, we performed super shift experiment (lanes 5 and 6). The presence of the HIF-1α protein in complex C1 was demonstrated, as the addition of anti-c-myc antibody (lane 6), but not a non-immune serum (lane 5), led to an up-shift of the complex and the formation of a new complex, C2. Anti-c-myc antibodies were unable to abolish binding of the complex C1 to the DNA completely, which led us to conclude that, in addition to HIF-1α, other proteins such as Sp1 are present in the C1 complex (lane 6). Competition using unlabelled wild type or mutant DNA probes verified the specificity of the complex (lanes 3 and 4).

**Figure 2:**
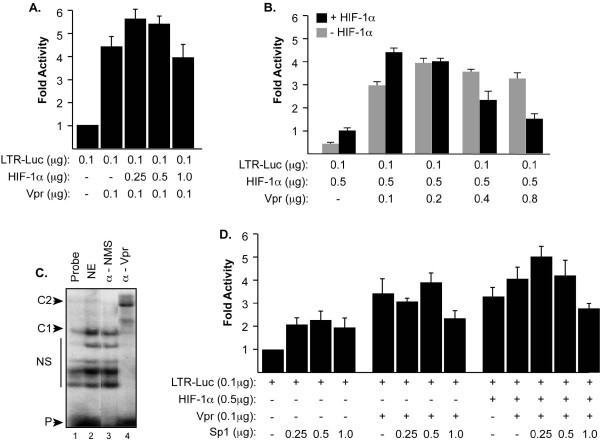
**Functional interplay between HIV-1 Vpr and HIF-1α**. ***A ***and ***B***. 786-O cells were transfected with LTR-Luc full length along with an increasing concentration of HIF-1α (A) and/or Vpr (B) expression plasmids. The amount of DNA in each transfection mixture was normalized with pcDNA_6_HisA. Luciferase activity was determined 48 hours after transfection. Results are displayed as histograms. ***C***. Approximately 100, 000 cpm of synthetic [γ^32^P]-labeled double-stranded DNA oligonucleotide probe corresponding to the HIV-1 LTR GC-rich site was incubated with 10 μg of nuclear extracts prepared from microglial cells transfected with Vpr expression plasmid. Labeled probe was also incubated with nuclear extracts prepared from pcDNA-transfected microglial cells in the presence of anti-Vpr antibody (lane 4) and normal mouse serum (NMS) (lane 2). ***D***. To further examine the interplay between Vpr and HIF-1α, 786-O cells were transfected with LTR-Luc along with HIF-1α, Vpr and/or Sp1 using different combinations as shown. Luciferase activity was determined 48 hours after transfection. Results are displayed as histogram. Each transfection was repeated 3 times using different DNA preps.

Previously, we demonstrated the ability of Vpr to modulate HIV-1 LTR transcription through its potential binding to the Sp1 transcription factor in the GC-rich region within the LTR [20]. To assess the effect of Vpr and/or Sp1 on transcription of the HIV-1 LTR in the presence and absence of HIF-1α, 786-O cells were transfected with 0.1 μg of LTR-luc together with 0.1 μg of Vpr expression plasmid in the presence of an increasing concentration of HIF-1α expression plasmid (0.25, 0.5 and 1.0 μg). As shown in Figure 2A, addition of HIF-1α did not affect the ability of Vpr to activate the LTR. Note that these cells do not express endogenous HIF-1α.

Reciprocally, we investigated the impact of an increasing concentration of Vpr on HIF-1α ability to activate the LTR. 786-O cells were transfected with a reporter plasmid containing the LTR upstream regulatory sequence fused to a reporter gene together with 0.5 μg plasmids expressing HIF-1α and/or increasing amount of Vpr. 786-O cells do not express endogenous HIF-1α [18]. As shown in Figure 2B, Vpr activates the LTR by 3-4 fold in the absence of HIF-1α. This activation was additively enhanced when 0.1 μg of Vpr and 0.5 μg of HIF-1α were co-expressed. Interestingly, activation of the HIV-1 promoter decreases when 0.8 μg of Vpr and 0.5 μg of HIF-1α plasmids were co-expressed leading to the conclusion that both proteins may be competing for Sp1 or LTR DNA interaction.

To further test this hypothesis, we performed gel shift assay using nuclear extracts from microglial cells, which we transfected with 5 μg of Vpr expression plasmids. Ten micrograms of nuclear extract prepared from the cells were incubated with labeled GC-rich dsDNA primer spanning nucleotides -82 to -61. As shown in Figure 2C, the intensity of the band corresponding to the DNA-Vpr complex was enhanced in cells transfected with a plasmid expressing Vpr (complex C1 in lane 2). To examine whether Vpr protein formed or was a part of complex C1, we performed super shift experiment (lanes 3 and 4). The presence of the Vpr protein in complex C1 was demonstrated, as the addition of anti-Vpr antibodies (lane 4), but not the addition of a non-immune serum (lane 3), led to an up-shift of the complex and the formation of a new complex, C2. Anti-Vpr antibodies were able to abolish binding of the complex C1 to the DNA completely, which led us to conclude that Vpr is the main protein present in the C1 complex (lane 4).

Finally, we examined whether Vpr and HIF-1α compete for Sp1 or LTR-DNA binding in transfection assays where we increased the concentration of Sp1. 786-O cells were transfected with LTR-luc in the presence and absence of constant amount of Vpr and/or HIF-1α expression plasmids along with an increasing concentration of Sp1 plasmid (0.25, 0.5 and 1.0 μg). As shown in Figure 2D, increasing amount of Sp1 plasmid had little or no effect on activation of the LTR by Vpr and/or HIF-1α when compared to the control.

**Figure 3:**
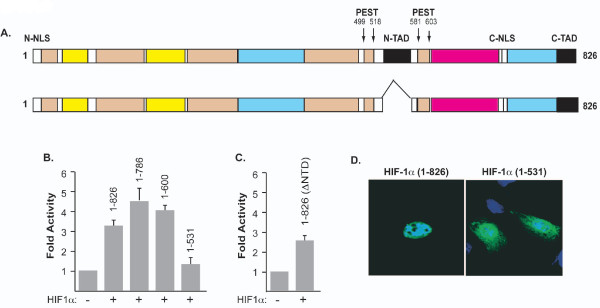
**Identification of the HIF-1α domain responsible for the LTR activation**. ***A***. Schematic representation of the structural organization of the HIF-1α domains. ***B ***and ***C***. 786-O cells were transfected with LTR-Luc along with HIF-1α full length and deletion mutant expression plasmids. The amount of DNA in each transfection mixture was normalized with pcDNA_6_HisA. Luciferase activity was determined 48 hours after transfection. Results are displayed as histograms. Each transfection was performed 3 times using different DNA prep. ***D***. Subcellular localization of HIF-1α. 786-O cells were transfected with c-myc-HIF-1α FL or deletion mutants as described in Methods section. Both proteins were detected in the nucleus and the cytoplasm (HIF-1α [1-531]) of the cells.

Next, we sought to identify the domain within HIF-1α responsible for HIV-1 LTR activation and interplay with Vpr. First, we examined the optimal concentration used by these mutants to activate the LTR and found that 0.5 μg of expression plasmids yielded the best activation (Figure 3B). Interestingly, we found that the region located between the 2 PEST domains that correspond to the N-transactivating domain of the HIF-1α protein may be the region responsible for the LTR activation.

To confirm this observation, we created a new deletion mutant of HIF-1α in which the N-TAD domain was deleted (Figure 3A). 786-O cells were transfected with 0.1 μg of the LTR-luc along with 0.5 μg of HIF-1 deletion mutant (ΔN-TAD). Twenty-four hours later, transfection was terminated and the cells were washed and processed for luciferase assay. As shown in Figure 3C, HIF-1α mutant modestly activated the LTR leading to the conclusion that additional domain(s) located in the N-terminal region of the HIF-1α may be involved in the LTR activation. The sub-cellular localization of HIF-1α mutants was also determined using immunocytochemsitry, and HIF-1α (full length and mutant) localized to the nucleus as well as the cytoplasm of the cells (HIF-1α [1-531]) (Figure 3D).

The functional interplay between Vpr and HIF-1α as well as the identification of the potential domain within HIF-1α responsible for activation of the LTR prompted us to examine the relation between Vpr and HIF-1α deletion mutants and their interplay in cells that lack HIF-1α. To that end, microglial cells were transfected with 0.1 μg of the LTR-luc along with 0.1 μg of Vpr and or 0.5 μg of HIF-1α (wt and deletion mutants) expression plasmids.

**Figure 4:**
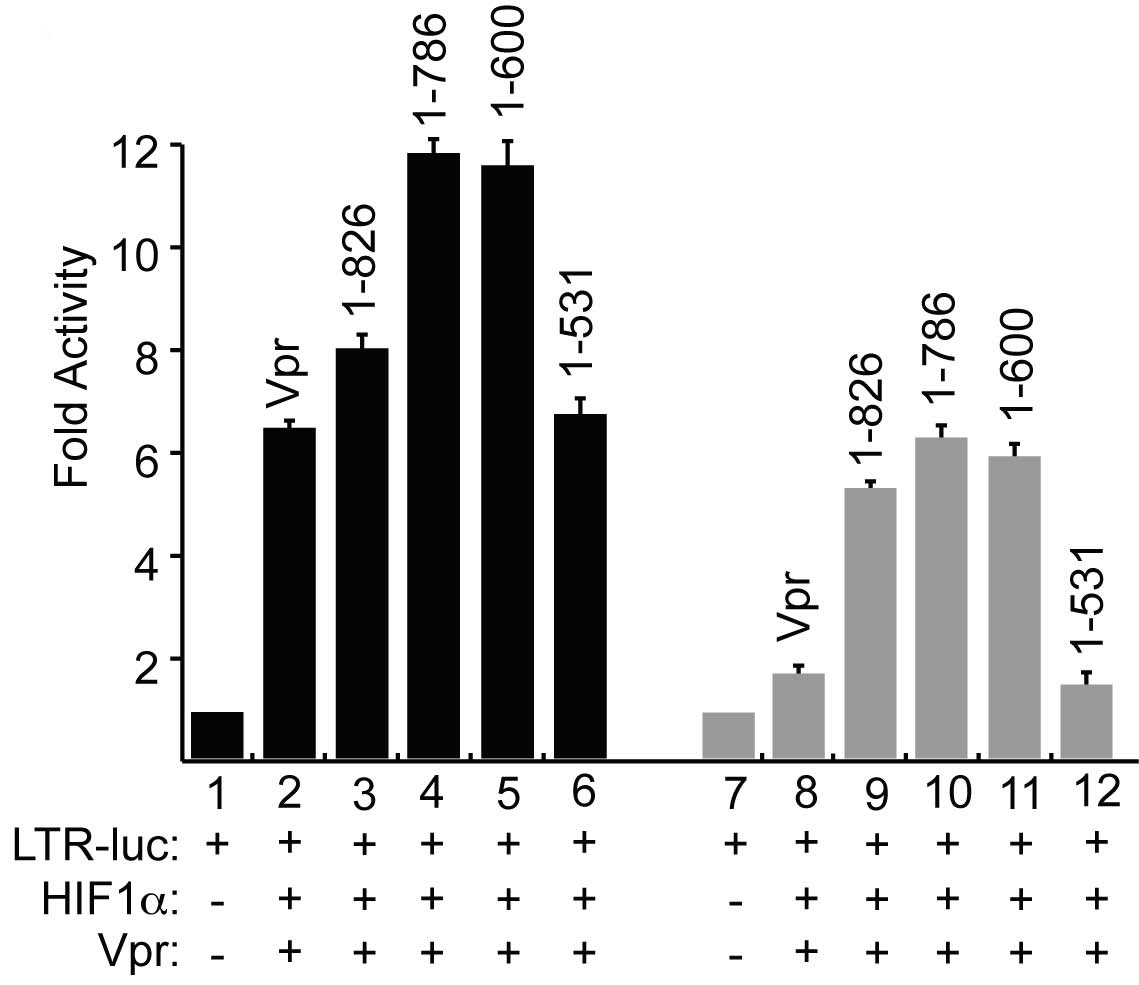
**Functional interplay between HIV-1 Vpr and HIF-1α in microglial and 786-O cells**. Microglial (lanes 1-6) and 786-O (lanes 7-12) cells were transfected with LTR-Luc full length along with HIF-1α full length and deletion mutant expression plasmids in the presence and absence of HIV-1 Vpr expression plasmids. The amount of DNA in each transfection mixture was normalized with pcDNA_6_HisA. Luciferase activity was determined 48 hours after transfection. Results are displayed as histograms. Each transfection was repeated 3 times using different DNA preps.

Twenty-four hours later, the cells were collected and processed for luciferase assay. Interestingly, while a modest additive activation of the LTR was observed in the presence of Vpr and full length HIF-1α (Figure 4A, lane 3), a significant additive effect was observed when Vpr was co-expressed along with HIF-1α mutants (lanes 4 and 5). Expectedly, HIF-1α mutant (1-531) did not affect the ability of Vpr to activate the LTR (lane 6).

A similar experiment was repeated using 786-O cells (HIF-1α^-/-^) and the results are displayed in lanes 7-12. Surprisingly, Vpr failed to activate the LTR in the absence of endogenous HIF-1α (lane 8). This activation was restored when Vpr was co-expressed with HIF-1α full length or deletion mutants (lanes 9-12). As expected, HIF-1α mutant (1-531), lacking both C-TAD and N-TAD did not affect the ability of Vpr to activate the LTR (lane 12). These results led us to conclude that Vpr requires a minimum presence of the HIF-1α in order to activate the HIV-1 LTR.

There is growing evidence that oxidative stress occurs in the brains of HIV-infected patients, and this can be one of the causes of progressive multiple symptoms of motor and cognitive dysfunction, and changes that characterize the AIDS dementia complex. Oxidative stress combined with activation of the inflammatory process plays a crucial role in disease progression in HIV-infected individuals. The complications in patients with AIDS are frequently a result of enhanced formation of many free radical species and the deregulation of endogenous antioxidant moieties. This suggests that, although essential for life, ubiquitous oxygen is also, by its products of metabolism, toxic to cells.

In this regard, HIF-1α was shown to be activated during cell stress and that this activation leads to its stabilization and activation of specific genes. The role of HIF-1α in cells infected with HIV-1 was unclear which provided us with the rationale to explore this role. To that end, we previously reported, that HIV-1 Vpr protein has the ability to promote stress in microglial cells leading to HIF-1α activation [6]. We also showed that activation of HIF-1α by Vpr leads to positive regulation of the LTR. Therefore, in this paper, we sought to examine the impact of HIF-1α up-regulation by Vpr on the HIV-1 promoter and Vpr functions. Interestingly, we demonstrated that Vpr activates the HIV-1 promoter through its functional interplay with HIF-1α and that this activation is lost in the absence of endogenous HIF-1α. Our data further confirm the need of Vpr to activate HIF-1α independently of the reactive oxygen species as we previously demonstrated [6]. It also point to the major role that HIF-1α plays during the regulation of the HIV-1 promoter.

These results led to the conclusion that HIF-1α might be playing the same role as APOBEC [21] and that will keep HIV-1 Vpr and Vif proteins, respectively, under control; a phenomenon that needs to be further studied in order to develop a Vpr inhibitor.

## Methods

### Plasmids

The HIF-1α plasmid (1-826) was previously described, while its deletion mutants (1/786; 1/600 and 1/531) were cloned using the following primers: (coding) 5'-ctggtt*ggatcc*gccaccatggagggcgccggcggcgcg-3' (*Bam*HI); (non coding) 786: 5'-aaccagat*gcggccgc*tcttgccccagcagtctaca-3'; 600: 5'- aaccagat*gcggccgc*tcaa ctgtgctttgaggact-5'; 531:5'-aaccagat*gcggccgc*tcattgaccatatcactatc-3' (*Not*I). Full length HIF-1α c-DNA was used as a template. The new products were cloned in-frame with Myc-His tag of pcDNA-6A plasmid. The internal deletion of N-terminal transactivation domain (Δ NTAD) was performed as previously described (Imai *et al*., 1991). Briefly, identification of the recombinant clone was done using a unique restriction site *Nhe*I, which was introduced in the place of deletion. The primers used were; (coding: 573/583) 5'- cgttccttcgatcagttgt cacca-3' and (non coding: 523/531) 5'-*gctagc*ttcattgaccatatcactatccac-3' (*Nhe*I).

The HIV-1 LTR and its deletion mutants as well as pcDNA_3_-Vpr (wt and mutant) and CMV- Sp1 plasmids were previously described [22,23].

### Cell Culture, and Transfection Assays

Human kidney (HIF-1α^-/- ^known as 786-O) [purchased from ATCC] and microglial cell lines were maintained in DMEM + 10% FBS. Cells were transfected with 0.1 μg of reporter plasmid (LTR-Luc) or co-transfected with 0.1-0.8 μg of Vpr or 0.5 μg of HIF-1α (wt or deletion mutants) expression cDNA. The amount of DNA used for each transfection was normalized with pcDNA_3 _vector plasmid. Each transfection was repeated multiple times with different plasmid preparations. Cell extracts were prepared 24 h after transfection, and Luciferase assay was performed as previously described [6].

### Gel electrophoresis mobility-shift assay (EMSA)

A gel electrophoresis mobility-shift assay was performed as described previously [22]. Oligonucleotides corresponding to the HIV-1 LTR promoter region were synthesized, annealed, labeled with [γ-^32^P] ATP and incubated at 4°C for 30 min with 10 μg nuclear extracts. For super shift assays, antibodies directed against Vpr were mixed with nuclear proteins for 1 h at 4°C prior to addition of the probe. The sequences of the oligonucleotides used in these experiments were 5'-tcccagggaggcgtggcctgg-3' (-82/-61) and 5'-ccaggccacgcctccct-3' (-61/-82).

### Immunocytochemistry

786-O cells were transfected with 1 μg of c-myc-HIF-1α full length or deletion mutant expression plasmids and seeded in poly-L-lysine-coated glass chamber slides. Cells were fixed in 4% paraformaldehyde, blocked with 5% normal horse serum in PBS-BSA 0.1% for 2 hrs and then incubated with anti-HIF-1α mouse (BD Bioscience) primary antibody over night. For HIF-1α mutant (1-531), anti-myc mouse primary antibody was used. Cells were then washed and incubated with FITC-conjugated anti-mouse secondary antibody for 1 hr. Expression of HIF-1α (FL and mutants) was visualized by fluorescence microscopy.

## Competing interests

The authors declare that they have no competing interests.

## Authors' contributions

SLD and SS carried out the molecular studies. SA, KK and BES designed the experiments, directed the work and analyzed the data. BES wrote the manuscript.

All authors read and approved the final manuscript.

## References

[B1] Pandey RC Datta D Mukerjee R Srinivasan A Mahalingam S Sawaya BE HIV-1 Vpr: a closer look at the multifunctional protein from the structural perspective Curr HIV Res 2009 7 114 128 10.2174/157016209787581508 19275580

[B2] Kitayama H Miura Y Ando Y Hoshino S Ishizaka Y Koyanagi Y Human immunodeficiency virus type 1 Vpr inhibits axonal outgrowth through induction of mitochondrial dysfunction J Virol 2008 82 2528 2542 10.1128/JVI.02094-07 18094160 PMC2258941

[B3] Sherman MP Greene WC Slipping through the door: HIV entry into the nucleus Microbes Infect 2002 4 67 73 10.1016/S1286-4579(01)01511-8 11825777

[B4] Amini S Khalili K Sawaya BE Effect of HIV-1 Vpr on cell cycle regulators DNA Cell Biol 2004 23 249 260 10.1089/104454904773819833 15142382

[B5] Jones GJ Barsby NL Cohen EA Holden J Harris K Dickie P Jhamandas J Power C HIV-1 Vpr causes neuronal apoptosis and in vivo neurodegeneration J Neurosci 2007 27 3703 3711 10.1523/JNEUROSCI.5522-06.2007 17409234 PMC6672409

[B6] Deshmane SL Mukerjee R Fan S Del Valle L Michiels C Sweet T Rom I Khalili K Rappaport J Amini S Sawaya BE Activation of the oxidative stress pathway by HIV-1 Vpr leads to induction of hypoxia-inducible factor 1alpha expression J Biol Chem 2009 284 11364 11373 19204000 10.1074/jbc.M809266200 PMC2670142

[B7] Simon MC Mitochondrial reactive oxygen species are required for hypoxic HIF alpha stabilization Adv Exp Med Biol 2006 588 165 170 10.1007/978-0-387-34817-9_15 17089888

[B8] Kupershmidt L Weinreb O Amit T Mandel S Carri MT Youdim MB Neuroprotective and neuritogenic activities of novel multimodal iron-chelating drugs in motor-neuron-like NSC-34 cells and transgenic mouse model of amyotrophic lateral sclerosis FASEB J 2009 23 3766 3779 10.1096/fj.09-130047 19638399

[B9] Lee DW Rajagopalan S Siddiq A Gwiazda R Yang L Beal MF Ratan RR Andersen JK Inhibition of prolyl hydroxylase protects against 1-methyl-4-phenyl-1, 2, 3, 6-tetrahydropyridine-induced neurotoxicity: model for the potential involvement of the hypoxia-inducible factor pathway in Parkinson disease J Biol Chem 2009 284 29065 29076 10.1074/jbc.M109.000638 19679656 PMC2781452

[B10] Ogunshola OO Antoniou X Contribution of hypoxia to Alzheimer's disease: is HIF-1alpha a mediator of neurodegeneration? Cell Mol Life Sci 2009 66 3555 3563 10.1007/s00018-009-0141-0 19763399 PMC11115623

[B11] Schults MA Timmermans L Godschalk RW Theys J Wouters BG van Schooten FJ Chiu RK Diminished carcinogen detoxification is a novel mechanism for hypoxia-inducible factor 1-mediated genetic instability J Biol Chem 2010 285 14558 14564 10.1074/jbc.M109.076323 20228066 PMC2863189

[B12] DeClerck K Elble RC The role of hypoxia and acidosis in promoting metastasis and resistance to chemotherapy Front Biosci 2010 15 213 225 10.2741/3616 20036816

[B13] Wang GL Semenza GL Purification and characterization of hypoxia-inducible factor 1 J Biol Chem 1995 270 1230 1237 10.1074/jbc.270.3.1230 7836384

[B14] Wang GL Jiang BH Rue EA Semenza GL hypoxia-inducible factor 1 is a basic-helix-loop-helix-PAS heterodimer regulated by cellular O_2 _tension Proc Natl Acad Sci USA 1995 92 5510 5514 10.1073/pnas.92.12.5510 7539918 PMC41725

[B15] Semenza GL Nejfelt MK Chi SM Antonarakis SE Hypoxia-inducible nuclear factors bind to an enhancer element located 3' to the human erythropoietin gene Proc Natl Acad Sci USA 1991 88 5680 5684 10.1073/pnas.88.13.5680 2062846 PMC51941

[B16] Maemura K Hsieh CM Jain MK Fukumoto S Layne MD Liu Y Kourembanas S Yet SF Perrella MA Lee ME Generation of a dominant-negative mutant of endothelial PAS domain protein 1 by deletion of a potent C-terminal transactivation domain J Biol Chem 1999 274 31565 31570 10.1074/jbc.274.44.31565 10531360

[B17] Carrero P Okamoto K Coumailleau P O'Brien S Tanaka H Poellinger L Redox-regulated recruitment of the transcriptional coactivators CREB-binding protein and SRC-1 to hypoxia-inducible factor 1alpha Mol Cell Biol 2000 20 402 415 10.1128/MCB.20.1.402-415.2000 10594042 PMC85095

[B18] Williams RD Elliott AY Stein N Fraley EE In vitro cultivation of human renal cell cancer. I. Establishment of cells in culture In Vitro 1976 12 623 627 10.1007/BF02797460 1010528

[B19] Jones KA Kadonaga JT Luciw PA Tjian R Activation of the AIDS retrovirus promoter by the cellular transcription factor, Sp1 Science 1986 232 755 759 10.1126/science.3008338 3008338

[B20] Sawaya BE Khalili K Mercer WE Denisova L Amini S Cooperative actions of HIV-1 Vpr and p53 modulate viral gene transcription J Biol Chem 1998 273 20052 20057 10.1074/jbc.273.32.20052 9685344

[B21] Romani B Engelbrecht S Glashoff RH Antiviral roles of APOBEC proteins against HIV-1 and suppression by Vif Arch Virol 2009 154 1579 1588 10.1007/s00705-009-0481-y 19669862

[B22] Amini S Saunders M Kelley K Khalili K Sawaya BE Interplay between HIV-1 Vpr and Sp1 modulates p21(WAF1) gene expression in human astrocytes J Biol Chem 2004 279 46046 46056 10.1074/jbc.M403792200 15302882

[B23] Sawaya BE Khalili K Gordon J Taube R Amini S Cooperative interaction between HIV-1 regulatory proteins Tat and Vpr modulates transcription of the viral genome J Biol Chem 2000 275 35209 35214 10.1074/jbc.M005197200 10931842

